# Evaluation of the Impact of a Psychoeducational Program on Type 1 Diabetes in Italian Schools: A Pre–Post Study

**DOI:** 10.1111/josh.70141

**Published:** 2026-03-30

**Authors:** Gaia Caldarelli, Alda Troncone, Antonietta Chianese, Angela Zanfardino, Serena Rollato, Giorgia Ippolito, Gulsum Ozen, Dario Iafusco

**Affiliations:** ^1^ Department of Psychology University of Campania “Luigi Vanvitelli” Caserta Italy; ^2^ Department of Woman, Child and General and Specialistic Surgery, Regional Center of Pediatric Diabetes University of Campania “L. Vanvitelli” Naples Italy; ^3^ Department of Pediatrics, Adolescent Health Ankara University Faculty of Medicine Ankara Turkey

**Keywords:** diabetes knowledge, diabetes psychoeducation, health education, school health, students, type 1 diabetes

## Abstract

**Background:**

Educating school personnel on Type 1 Diabetes (T1D) management is essential for ensuring prompt interventions, preventing complications, and creating a safe environment for students. This study evaluated the impact of a synchronous, online psychoeducational program implemented in schools in Southern Italy.

**Contributions to Practice:**

This research demonstrates the positive impact of a psychoeducational intervention program aimed at increasing T1D literacy, developed in accordance with national and international guidelines and delivered in schools attended by children receiving care at the authors' clinical center. Pre–post questionnaires completed by 436 staff members indicated significant increases in T1D knowledge (*p* < 0.001, *d* = 0.48) and perceived self‐efficacy (*p* < 0.001, *d* = 0.99), as well as improved ability to recognize hypoglycemia symptoms (*p* < 0.001, *d* = 0.97). Additionally, the program reduced dysfunctional beliefs regarding the needs of children and adolescents with T1D (*p* < 0.001).

**Implications for School Health Policy, Practice, and Equity:**

Implementing a T1D psychoeducational program for school staff members may promote the inclusion of students with T1D and help dispel misconceptions about the condition and students' care needs.

**Conclusions:**

This study provides evidence of the positive impact of a synchronous online program to improve T1D knowledge and perceived self‐efficacy among Italian school staff.

## 1 Introduction

Type 1 diabetes (T1D) is characterized by hyperglycemia resulting from the autoimmune destruction of pancreatic beta cells, leading to a deficiency or complete absence of endogenous insulin. The International Diabetes Federation (IDF) estimates that 537 million people are living with diabetes globally, with more than 1.2 million children and adolescents affected by T1D specifically [1]. A T1D regimen requires strict adherence to structured plans, which include pharmacological treatment (i.e., insulin therapy), controlled dietary habits (i.e., managing carbohydrate intake), and regular physical activity [2]. It also involves continuous monitoring of blood glycemic levels and requires prompt intervention in cases of hypoglycemia or hyperglycemia. The introduction of new technologies (including artificial pancreas systems, insulin pumps, and continuous glucose monitoring [CGM] sensors) in recent years has led to more sophisticated and demanding T1D management [3]. Consequently, T1D management activities must be carried out in children's daily environments, such as schools [4].

Research has shown that poor T1D management can impact not only physical health but also academic performance; children with poor metabolic control have poorer reading skills and lower average school grades than their peers [5]. Increased absenteeism was also found in children with T1D due to poor glycemic control and diabetes management routines, which are often carried out during school hours [6]. Conversely, Dahlquist et al. [7] found that minimizing episodes of hypoglycemia and hyperglycemia by effectively managing T1D improves the academic achievement of children with T1D to a level comparable to that of their peers.

It has therefore become crucial to educate school staff on diabetes management practices to ensure prompt interventions and prevent short‐ and long‐term diabetes complications, while also creating a safe environment for children with T1D [4].

Recent international studies have investigated teachers' perceptions and understanding of the disease and its management, finding that most had only basic knowledge of T1D's characteristics and related emergency management procedures [8, 9]. A study conducted in Saudi Arabia, which has the 4th highest T1D rates globally, found that only 10.8% of interviewed teachers had attended a course on T1D, and fewer than 32.5% were aware of specific school programs for managing T1D [8].

In Italy, diabetes is estimated to affect more than 3.5 million people [10], with 2357 cases of T1D reported among individuals aged 0–29 years between 2000 and 2012 [11]. The needs of Italian students with T1D are addressed by the “Linee Guida per la Somministrazione dei Farmaci in Orario Scolastico” (Guidelines for the Administration of Medication During School Hours) [12], which stipulates that teachers may administer glucagon^1^ if prescribed by a doctor and if parents submit a formal request. These guidelines also strongly encourage the presence of teachers or other school staff trained in diabetes management [12]. If such personnel are unavailable or if proper storage of emergency medication is not possible, an agreement should be established with the local assistance service [12]. However, parents of Italian children with T1D have reported that schools are ill‐prepared to manage their children's diabetes, citing difficulties in finding staff willing to administer glucagon or other medication [13, 14]. In another study, teachers confirmed that they had received no specific training in diabetes management [14]. This research suggests a clear need to provide teachers with additional information and practical training to effectively manage diabetic emergencies.

Despite previous research indicating that diabetes‐related training enhances school staff knowledge and confidence in managing diabetes [15, 16, 17, 18], few Italian studies have evaluated diabetes education programs and their effectiveness. To the authors' knowledge, only one Italian study has assessed the efficacy of an online program for school staff members, reporting that individuals who attended the training demonstrated better theoretical knowledge of diabetes than those who did not [19].

This study evaluated the impact of a synchronous online psychoeducational intervention designed to increase T1D literacy among school staff, conducted in schools in Southern Italy.

We hypothesized that participating in this program on T1D could:

– Enhance the theoretical knowledge of school staff.
– Reduce stigma and misconceptions regarding T1D management and students living with T1D.
– Increase school staff members' self‐perceived efficacy in managing T1D, leading to more prompt and effective assistance for students.

## 2 Methods

### 2.1 Description of the Psychoeducational Program

The intervention was designed in accordance with the international guidelines from the International Society for Pediatric and Adolescent Diabetes (ISPAD) and the national guidelines from the Associazione Giovani con Diabete Italia (AGDI [Association of Young People with Diabetes in Italy]). These bodies emphasize the importance of educating healthcare workers, teachers, and sports coaches in T1D management and the prevention and handling of emergencies [20, 21]. The AGDI guidelines emphasize that effective integration of children with T1D into primary and secondary schools requires a coordinated plan involving schools, families, and local health centers [20]. ISPAD highlights that T1D education and awareness can facilitate the psychosocial integration of children with the condition by reducing prejudice among teachers and classmates [21]. We therefore developed a psychoeducational session to increase knowledge among school staff and promote the integration of students with T1D.

The intervention comprised a single module led by a diabetologist and a psychologist, who provided information about T1D and its management. Several schools were invited to participate and were grouped by educational level (i.e., primary, middle, and high school). Given that prior studies have demonstrated positive effects of online programs [16; 18], and to increase the number of schools involved and facilitate staff participation, the intervention was delivered in a synchronous and online format. Each training session lasted approximately 1 h and was delivered via the Google Meet platform.

The module covered topics including the definition of T1D, its clinical signs and symptoms, the management of diabetes at school, and dealing with emergencies that may occur during school hours. Additionally, the intervention emphasized the emotional challenges faced by children and adolescents with T1D and their relationships with their peers. Because children at different ages and developmental stages experience distinct challenges, the psychoeducational program was tailored to the specific needs of each grade level. For instance, the session with high school teachers addressed the challenges of adolescence, such as difficulties in peer relationships and the desire to avoid T1D‐related restrictions. Participants were given the opportunity to discuss their concerns or issues that had arisen in their classrooms. Parents were also invited to attend the session as observers and, if necessary, to express and discuss their concerns with the aim of improving the management of their child's T1D at school.

### 2.2 Procedure and Data Collection

This study recruited schools in Southern Italy attended by children receiving care at the authors' clinical center. In accordance with ADGI guidelines, following a child's first hospitalization at the clinical center (and initial diagnosis of T1D), a clinical report was provided to the school and teachers to support the student's reintegration into the school environment. Once the child had returned to school, the clinical center contacted the school to assess its willingness to participate in the psychoeducational program. Access to the module depended on the presence of at least one child with T1D at the school. However, all school staff members (principals, teachers, and nonteaching staff) from the contacted schools were eligible to participate. The scheduled date for the intervention was communicated to the school's designated representatives, who disseminated the initiative to the staff, who could then decide whether to participate in the program. A member of the clinical center contacted the school a few days later to request the list of participants in order to send them the first questionnaire link and, upon completion of the program, the certificate of attendance.

The study employed a pre–posttest design without a control group. School staff members who registered on the participation list were asked to complete a questionnaire assessing their knowledge, attitudes, concerns, and beliefs regarding T1D prior to participating in the intervention. Data were collected using an anonymous online questionnaire hosted on Google Forms, administered to school staff members before the intervention and immediately following its completion. The link to the questionnaire was sent to the school's administrative team or the teachers' coordinator, who then distributed it to the participants.

At the end of the intervention, the link to the posttest questionnaire was shared via the platform's chat, and participants were asked to complete it. To ensure that the questionnaire was also accessible to those who were unable to complete it during the session, the link was subsequently sent via email, along with a booklet summarizing the topics covered during the session.

Data were collected from September 2023 to December 2024. All school staff members provided written, digital informed consent. All procedures were authorized by the ethics committee of the authors' institution (approval no. 4/01.03.2022) and conducted in accordance with the ethical principles of the Declaration of Helsinki.

### 2.3 Measures

The study questionnaire was developed following a thorough review of the relevant literature [22, 23, 24, 25, 26, 27, 28]. Questionnaire items and their corresponding references are provided in Table S1. The questionnaire comprises four sections, including a total of 24 questions covering: (1) demographic and general information, (2) knowledge of scholastic personnel about T1D, (3) attitude toward worries and beliefs regarding T1D, and (4) self‐perceived competence regarding diabetes knowledge and managing T1D. For “Section 2: Knowledge of Type 1 Diabetes,” one point was awarded for each correct answer, for a total of 11 points.

The questionnaire's content validity was assessed by a group of T1D patients and care providers, including a physician, a diabetes educator nurse, a diabetes educator dietitian, three pediatric diabetes experts, two psychologists with expertise in diabetes, and a teacher. This process ensured the items were consistent with current diabetes management and practices. In line with the experts' suggestions, one item was revised for grammatical accuracy (item 1, Section 4), and one was modified to clarify content (item 3, Section 2).

### 2.4 Data Analysis

Responses to questions in Sections 2, 4 were presented as frequencies and analyzed using percentage increases (i.e., proportional percentage changes; calculated as: post−pre/pre × 100) to identify changes in staff perceptions regarding T1D following the intervention.

To detect differences between categories (i.e., between school staff members completing both assessments and those completing the preassessment only, as well as between pre‐ and postintervention answers to Sections 2, 4), we employed the Pearson chi‐square test for independent samples and the McNemar–Bowker test for repeated categorical measures for categorical variables, and an independent‐samples or a paired‐sample *t* test for continuous variables. Where the Pearson *χ* ^2^ test identified significant differences, we examined adjusted standardized residuals (ASRs) to identify observed values that differed significantly from expected values (> |2|). Effect sizes were reported as Cohen's *d* (*d* = 0.10: small; 0.50: medium; 0.80: large) [29]. All analyses were performed using Microsoft Excel and SPSS version 26.0 for Macintosh.

## 3 Results

### 3.1 Participants

Of the 70 schools that received the clinical reports, 61 institutions across Southern Italy requested the intervention, ranging from kindergarten to high schools. Of these, the majority (*n* = 59) were located in the Campania region (covering all regional districts except Benevento), with 1 primary school in the Sicily region and 1 primary school in the Lazio region.

Of the 670 school staff members enrolled in the first phase of the study, 234 (34.97%) only completed the preintervention assessment. Among these, 20 participants were unable to complete the posttest questionnaire due to technical issues, and 214 were registered on the participant list but were unable to attend the session due to concurrent work‐related meetings or temporary personal issues. Ultimately, 436 school staff members attended the T1D lesson and completed both pre‐ and postassessments; all responded to all items, resulting in no missing data.

Table 1 presents the socio‐demographic characteristics of the final sample. Most participants were female (87.8%), had a university degree (68.8%, including both bachelor's and master's degrees), and worked in a public school (97.7%). On average, participants had worked at the school for almost 18 years, and 63.1% had a child or youth with T1D in their class.

**TABLE 1:** Sociodemographic characteristics of the sample (answers to first section of questionnaire).

	Whole sample *N* = 436
Gender (female) (*n*, %)	383 (87.8)
Age (years) M(SD)	50.75 (±10.03)
Years working in the school *M* (SD)	18.11 (±11.5)
Level of education	
Middle school graduation	1 (0.2)
High school graduation	99 (22.7)
Degree	300 (68.8)
Postlauream degree	36 (8.3)
Position held in the school (*n*, %)	
Principal	4 (0.9)
Teacher	420 (96.3)
Nonteaching staff	11 (2.5)
Not declared	1 (0.2)
Type of school (*n*, %)	
Public	426 (97.7)
Private	10 (2.3)
Diabetic children in the class (*n*, %)	
Yes	275 (63.1)
No	161 (36.9)

To mitigate potential attrition bias, a comparative analysis was conducted between participants who completed both the pre‐ and postintervention questionnaires and those who completed only the preintervention questionnaire. We found no significant differences in gender, age, years of experience in the school, type of school, or the presence of children with T1D in the class (Table S2). However, those who did not complete the postassessment were more likely to have a middle‐school education level (*p* = 0.027; ASR: +2.8) or be nonteaching staff (*p* = 0.007; ASR: +3.3), and less likely to be teachers (*p* = 0.007; ASR: −2.7). Baseline knowledge of diabetes (Section 2 of the questionnaire) did not differ significantly between the two groups.

### 3.2 Outcomes of the Intervention on T1D Knowledge

During the preintervention phase, participants scored an average of 9.66 out of 11 points on general knowledge about T1D characteristics (Table 5). Following the intervention, the number of correct answers increased, reflecting a significant improvement in overall T1D knowledge (average 10.32, *p* < 0.0001, *d* = 0.48) (Table 5). Specifically, school staff demonstrated improved knowledge of diabetes treatment and an improved ability to recognize and manage symptoms of hypoglycemia and hyperglycemia (all *p* < 0.001). However, no significant changes were observed for questions no. 1 (What is diabetes disease?; *p* = 0.85), no. 2 (Is diabetes a contagious disease? *p* not calculated, as there was no variance in the answers), and no. 3 (What are the symptoms of diabetes? *p* = 0.72) (Table 2).

**TABLE 2:** Answers to second section “Knowledge of Type 1 Diabetes.”

*n*	Whole sample *N* = 436	Preintervention answers (*n*, %)	Postintervention answers (*n*, %)	Proportionalpercentage increase (%)	McNemar‐Bowker test, *p*
1	What is diabetes disease?				2.67, 0.85
	A kidney disease	2 (0.5)	4 (0.9)	80	
	The body has not enough calcium	0	2 (0.5)	n/d	
	Lack of oxygen	0	0	—	
	The body has not enough insulin	434 (99.5)	430 (98.6)	−0.9	
2	Is diabetes a contagious disease?				n/d
	Yes	0	0	—	
	No	436 (100)	436 (100)	—	
	I don't know	0	0	—	
3	What are the symptoms of diabetes?				3.67, 0.72
	Autism	0	0	—	
	Excessive thirst and urge to urinate	430 (98.6)	435 (99.8)	1.22	
	Bleeding and dizzy	5 (1.1)	1 (0.2)	−81.82	
	High fever and sore throat	1 (0.2)	0	−100	
4	Type 1 diabetes is treated with:				28.33, < 0.001
	A low‐sugar diet	26 (6)	10 (2.3)	−61.67	
	Insulin	51 (11.7)	72 (16.5)	41.03	
	Insulin and a low‐sugar diet	343 (78.7)	353 (81)	2.92	
	I don't know	16 (3.7)	1 (0.2)	−94.59	
5	What does hypoglycemia mean?				20.12, < 0.001
	High blood sugar	31 (7.1)	13 (3)	−57.75	
	Low sugar in blood	398 (91.3)	418 (95.9)	5.04	
	Sugar in urine	1 (0.2)	5 (1.1)	450	
	I don't know	6 (1.4)	0	100	
6	Which of the following are considered as low blood sugar sign?				31.29, < 0.001
	Hot flashes, itching, diarrhea	0	0	—	
	Need to urinate, thirst, vomiting	49 (11.2)	56 (12.8)	14.29	
	Paleness, sweating, shakiness	350 (80.3)	376 (86.2)	7.35	
	I don't know	37 (8.5)	4 (0.9)	−89.41	
7	Hypoglycemia could be handled at school				25.36, < 0.001
	Yes, from teachers	59 (13.5)	46 (10.6)	−21.48	
	Yes, only by 911	12 (2.8)	4 (0.9)	−67.86	
	No	15 (3.4)	1 (0.2)	−94.12	
	Yes, from the child with T1D and teachers	350 (80.3)	385 (88.3)	9.96	
8	What is the right procedure when someone has a low blood sugar?				42.72, < 0.001
	Give him/her glucose strip or sugar drink.	381 (87.4)	429 (98.4)	12.61	
	Give him/her a cup of water.	9 (2.1)	0	−100	
	Give him/her chocolate.	10 (2.3)	1 (0.2)	−91.30	
	Give him/her biscuits or sweets	36 (8.3)	6 (1.4)	−83.13	
9	Which of the following is considered as high blood sugar signs?				39.22, < 0.001
	Frequent urination and thirstiness	353 (81)	400 (91.7)	13.21	
	Irritability	15 (3.4)	12 (2.8)	−17.65	
	Hyperactivity	18 (4.1)	17 (3.9)	−4.88	
	I don't know	50 (11.5)	7 (1.6)	−86.96	
10	Is it necessary for a child/adolescent with type 1 diabetes to have a mid‐morning snack?				63.57, < 0.001
	Yes	355 (81.4)	422 (96.8)	18.91	
	No	10 (2.3)	9 (2.1)	−8.7	
	I don't know	71 (16.3)	5 (1.1)	−93.34	
11	A child/adolescent with type 1 diabetes at school should				32.8, < 0.001
	Avoid frequent blood glucose checks or eating snacks outside scheduled times	28 (6.4)	19 (4.4)	−31.25	
	Participate fully in all school activities, with the freedom to self‐monitor and consume necessary foods	380 (87.2)	415 (95.2)	9.17	
	Avoid participating in physical education classes	0	1 (0.2)	n/d	
	I don't know	28 (6.4)	1 (0.2)	−96.88	

### 3.3 Outcomes of the Intervention on T1D Attitudes, Worries, and Beliefs

Participants' responses to questions in the “Attitudes, worries and beliefs on T1D” section prior to receiving the intervention indicated that they perceived diabetes as having a relatively limited impact on students' achievement, behavior, and psychological needs. After the intervention, all responses to questions in this section showed statistically significant changes (all *p* < 0.05; Table 3). Notably, the proportion of “I don't know” answers decreased significantly, indicating greater awareness of the effects of T1D on students' daily lives and specific needs. In particular, following the intervention, “I don't know” responses decreased for questions no. 2 (Does a child/adolescent with T1D miss more school than their classmates?) (−70.33%), no. 3 (I realize that diabetes can affect students' ability to pay attention in lessons) (−77.78%), no. 4 (I consider diabetic students as special needs students) (−70.89%), and no. 5 (Children/adolescents with T1D may have more behavioral problems) (−80.67%). We also observed an increase in the belief that having information on T1D would improve children's integration at school (*p* < 0.001) (Table 3).

**TABLE 3:** Answers to third section “Attitude toward T1D, worries and beliefs on T1D.”

*n*	Whole sample *N* = 436	Preintervention answers (*n*, %)	Postintervention answers (*n*, %)	Proportional percentage increase (%)	McNemar‐Bowker test, *p*
1	Diabetes has an impact on the student's achievement				56.33, < 0.001
	Yes	86 (19.7)	101 (23.2)	17.77	
	No	280 (64.2)	326 (74.8)	16.51	
	I don't know	70 (16.1)	9 (2.1)	−86.96	
2	Does a child/adolescent with type 1 diabetes miss more school than their classmates?				45.55, < 0.001
	Yes	29 (6.7)	30 (6.9)	2.99	
	No	316 (72.5)	379 (86.9)	19.79	
	I don't know	91 (20.9)	27 (6.2)	−70.33	
3	I realize that diabetes can affect students' ability to pay attention in lessons.				56.23, < 0.001
	Yes	172 (39.4)	193 (44.3)	12.43	
	No	166 (38.1)	221 (50.7)	33.1	
	I don't know	98 (22.5)	22 (5)	−77.78	
4	I consider diabetic students as special needs students				43.49, < 0.001
	Yes	126 (28.9)	112 (25.7)	−11.07	
	No	241 (55.3)	304 (69.7)	26.04	
	I don't know	69 (15.8)	20 (4.6)	−70.89	
5	Children/adolescents with type 1 diabetes may have more behavioral problems				64.46, < 0.001
	Yes	43 (9.9)	47 (10.8)	9.09	
	No	300 (68.8)	371 (85.1)	23.7	
	I don't know	93 (21.3)	18 (4.1)	−80.67	
6	Students with type 1 diabetes make problems with their friends.				10.30, 0.016
	Yes	28 (6.4)	34 (7.8)	21.88	
	No	356 (81.7)	383 (87.8)	7.47	
	I don't know	52 (11.9)	19 (4.4)	−63.03	
7	Do you think that more information about type 1 diabetes would improve children's integration at school?				34.046, < 0.001
	Yes	398 (91.3)	415 (95.2)	4.27	
	No	18 (4.1)	16 (3.7)	−9.76	
	I don't know	20 (4.6)	5 (1.1)	−76.09	

### 3.4 Outcomes of the Intervention on Self‐Perceived Competence About One's Own Knowledge and Abilities in Managing T1D

Participants' responses to the question asking them how they would feel about teaching a child with T1D showed significant changes following the intervention (*p* < 0.001; Table 4). As in the previous section, the proportion of “I don't know” responses decreased significantly (−69.81%), reflecting a reduction in the proportion of school staff reporting feeling anxious or confused about teaching or caring for a child with T1D and an increase in those describing themselves as relaxed (+32.7%).

**TABLE 4:** Answers to fourth section “Self‐perceived competence about one's own knowledge and abilities in managing T1D.”

*n*	Whole sample *N* = 436	Preintervention answers (*n*, %)	Postinterventionanswers (*n*, %)	Proportionalpercentage increase (%)	McNemar‐Bowker test, *p*
1	How would you personally feel about teaching a child with T1D?				47.44, < 0.001
	Anxious, nervous	67 (15.4)	47 (10.8)	−29.87	
	Relaxed	307 (64.2)	371 (85.1)	32.7	
	Confused	16 (3.7)	4 (0.9)	−75.68	
	I don't know	46 (10.6)	14 (3.2)	−69.81	
2	In your opinion, is it helpful or reassuring for the parent to be able to monitor their child with type 1 diabetes from home and intervene at any time by calling and asking for corrections to be made?				12.67, 0.05
	Helpful, reassuring	368 (84.4)	385 (88.3)	4.63	
	Annoying, a source of anxiety	19 (4.4)	24 (5.5)	25	
	Intrusive	5 (1.1)	6 (1.4)	27.27	
	I don't know	44 (10.1)	21 (4.8)	−52.48	

No significant changes were observed in participants' opinions regarding parental monitoring of children's diabetes (question no. 2, *p* = 0.05; Table 4), which they described as helpful in both the pre‐ and postintervention phases. Notably, the number of participants who considered parental monitoring “annoying” or “intrusive” increased, likely due to the reduction in “I don't know” responses (−52.48%) in the postintervention phase (Table 4).

Regarding participants' perceptions of their overall knowledge and abilities in T1D management, they demonstrated a significant increase in their perceived knowledge (*p* < 0.0001, *d* = 0.95), management abilities (*p* < 0.0001, *d* = 0.99), and ability to recognize symptoms of hypoglycemia (*p* < 0.0001, *d* = 0.97) following the intervention (Table 5). Furthermore, after the intervention, a significant increase was observed in the extent to which teachers perceived parental interference in the school lives of children with T1D (*p* = 0.009, *d* = 0.13).

**TABLE 5:** Comparison of the means for the second section's answers (sum) and for the fourth section's answers (Likert scale).

*n*	Whole sample *N* = 436	Preintervention (M, SD)	Postintervention(M, SD)	*t* test	*p*	*d*
—	Sum of answers of Second section	9.66 (±1.35)	10.32 (±0.851)	−10.01	0.000	0.48
3	How would you rate your knowledge about diabetes?	5.46 (±1.81)	7.09 (±1.31)	−19.88	0.000	0.95
4	How would you rate your ability in diabetes management?	5.01 (±1.94)	6.85 (±1.35)	−20.68	0.000	0.99
5	How would you rate your ability to recognize symptoms of low blood glucose levels?	5.06 (±1.93)	6.91 (±1.41)	−20.21	0.000	0.97
6	How much do you think a parent of a child/adolescent with type 1 diabetes can interfere with the child's school life?	5.46 (±2.27)	5.75 (±2.12)	−2.64	0.009	0.13

## 4 Discussion

This study illustrates the impact of a psychoeducational program developed to increase T1D literacy among school staff. To the authors' knowledge, this study is the first to involve schools from three different Italian regions and include a large sample of teachers, principals, and other school personnel. Despite a relatively high attrition rate (35%), the substantial similarity between completers and noncompleters mitigates—although does not entirely eliminate—the potential for attrition bias, indicating that the findings remain generalizable and can be meaningfully interpreted.

Our results indicate that school staff possessed an acceptable level of knowledge about diabetes prior to the intervention. This finding enriches previous evidence, which often presents a conflicting picture, with several studies reporting poor diabetes knowledge among teachers and other nonclinical school staff [15, 16, 23] and others finding that while teachers had sufficient knowledge about general aspects of diabetes, they lacked expertise regarding specific complications and management strategies [30]. In the context of the present study, the observed acceptable levels of baseline knowledge may be attributed to the basic nature of the items, which focused on common knowledge about T1D, and/or to staff members' prolonged experience in the school setting (with participants reporting an average of 18 years working in schools), which increases the probability that they had prior experience managing situations involving childhood and adolescent illnesses, such as T1D. Nevertheless, consistent with previous findings [19], we observed a significant increase in overall T1D knowledge following the psychoeducational program, confirming the first hypothesis.

Additionally, in line with the second hypothesis, implementing a program on T1D was found to enhance staff understanding of the impact of diabetes on children's behavior at school and of the challenges these students face. Specifically, we observed greater overall awareness of how T1D may or may not affect students' daily lives and specific needs, suggesting that the intervention improved the ability of school staff to recognize the needs of students with T1D and to avoid misinterpreting them as behavioral or psychological problems, thereby reducing prejudice toward their condition [18]. Supporting this assumption, approximately 85% of participants (a 33% increase) reported feeling more confident in teaching and caring for a student with T1D following the intervention.

Notably, the analysis of participant responses also elucidated school staff members' opinions regarding the utility of parental monitoring of children with T1D during school hours. School personnel described such monitoring as both helpful and intrusive. Interestingly, the perceived intensity of parental interference in the school lives of children with T1D increased following the intervention. This perception may stem from the pressure that teachers often feel from parents who are understandably concerned about diabetes‐related risks, such as hypoglycemia, during school hours. More broadly, aspects of diabetes care, such as parents' ability to remotely monitor their child's blood glucose levels via a mobile phone, may intensify teachers' sense of being monitored or pressured, contributing to a perception of parental intrusiveness. While remote monitoring during school hours may reduce parental anxiety [31, 32], the availability of real‐time data and the possibility of prompt action may lead some parents to contact staff more frequently, potentially creating the perception that school personnel are under continuous supervision. This dynamic may undermine teachers' sense of professional autonomy or competence in managing routine school situations involving students with T1D and, consequently, contribute to the perception of parental intrusiveness. In the context of the intervention, it could also be hypothesized that the mere presence of parents at the session, even when silent, may have contributed to teachers' perception of parental intrusiveness.

Overall, these results highlight the importance of holding regular meetings to facilitate cooperation between schools and families, clarify aspects of T1D management, and ensure the full inclusion of children with T1D, while avoiding prejudice toward certain behaviors and minimizing perceptions of parental intrusiveness.

Consistent with the third hypothesis, participant responses showed an increase in self‐perceived efficacy in managing T1D following the intervention. This is likely attributable to their improved knowledge of T1D characteristics and management strategies. It is reasonable to assume that the opportunity to interact with T1D experts (i.e., diabetologists, psychologists, dietitians) during the educational session, as well as the chance to discuss doubts or concerns with the group, may have also increased perceived self‐efficacy.

Overall, consistent with previous research [15, 17, 18, 19, 33], our findings highlight the positive impact of a psychoeducational diabetes program. As noted by several studies, a psychoeducational program on diabetes for school personnel is useful for ensuring the prompt assistance and management of students with diabetes, especially those in kindergarten and primary school [18, 34, 35].

### 4.1 Limitations and Future Directions

This study presents several limitations. First, the voluntary nature of participation, linked to the presence of a student with T1D in the class, may have introduced self‐selection bias, potentially excluding individuals who were interested but chose not to participate. Furthermore, the relatively high rate (35%) of noncompletion among school staff, despite minimal baseline differences, may have introduced attrition bias and potentially led to an overestimation of the program's impact, thereby limiting the generalizability of these findings. The absence of a control group may have precluded causal inferences about the effects of the psychoeducational program.

Additionally, the evaluation was conducted immediately after the intervention, and no assessment of long‐term retention was performed; this may have contributed to an overestimation of short‐term effects and did not account for the realistic impact of a 1‐h program. Furthermore, although the educational program was based on national recommendations, it was implemented and applied for the first time, which may limit its applicability to broader contexts. It should also be noted that some cells in the McNemar–Bowker test had frequencies below 5, which could have influenced or reduced the test's robustness, necessitating caution when interpreting and generalizing these findings.

As this was a first‐program implementation, some questions in the questionnaire warrant further refinement. In particular, questions no. 1 and no. 2 in Section 4 may have introduced bias by forcing respondents to choose among predefined adjectives to describe their feelings about teaching a child with T1D and their perception of parental monitoring, respectively. Adopting a Likert‐scale format in future versions may enhance objectivity and reduce interpretative constraints. Finally, the use of a self‐report instrument may have introduced social desirability bias, especially in the postassessment for items assessing self‐perceived competence.

### 4.2 Implications for School Health Research

Future research would benefit from employing a larger sample size, expanding the intervention to regions of Northern and Central Italy, and including a control group to enhance the validity and generalizability of the results. A follow‐up study could be conducted to assess teachers' knowledge and perceptions of diabetes to evaluate the long‐term effects of the educational program and its actual effectiveness despite its 1‐h duration. Additionally, analyzing students' and families' perceptions after the intervention could provide a deeper understanding of the program's impact on school diabetes management for both teachers and students. Future studies could also adopt a standardized educational protocol across all Italian schools to increase the generalizability of the findings and facilitate comparison among institutions. To further improve the protocol, future developments may include the option to deliver the intervention online or in person, according to the needs and constraints of school staff. Such flexibility would also allow for a systematic examination of the role of delivery modality in influencing intervention effectiveness. A supplementary follow‐up coaching session offered to teachers with a T1D student in their classrooms could be implemented to strengthen the long‐term retention of the program's effects. Future research should also investigate the impact of parental involvement during intervention sessions, paying particular attention to whether an active, participatory role—rather than mere attendance—may be associated with differences in intervention effectiveness.

### 4.3 Implications for School Health Policy, Practice, and Equity

According to the literature, these findings highlight the importance of cooperation between local health services and schools through the implementation of an accessible program for all school staff [15, 36]. Attending a brief online intervention involving diabetes experts increased school workers' understanding of their pupils' needs. Furthermore, the skills acquired by school staff may facilitate prompt interventions, thereby reducing the risks and consequences associated with hypo‐ or hyperglycemia and enabling early recognition of some diabetes‐associated symptoms.

Considering these and prior findings, establishing national guidelines for diabetes education programs that target all school staff could improve the quality of life for students with T1D by creating a safe environment in which they can receive personalized support. Furthermore, it is crucial that teachers are trained to support their students with T1D. This could help to ensure the full inclusion of these students in school activities and to mitigate erroneous beliefs regarding children with T1D and their needs. This is particularly important in light of substantial evidence indicating an increased risk of psychological difficulties among youth with T1D, including depressive and anxiety symptoms, body image concerns, and heightened psychological distress, which may interfere with psychosocial development and adversely affect school performance [37, 38, 39].

Given the positive outcomes observed in this study, this intervention could be incorporated into routine school–health collaboration frameworks or regional education policies, thereby supporting its implementation in real‐world settings and enhancing its potential impact on the well‐being of students and teaching staff.

## 5 Conclusion

This study provides evidence of the positive impact of a synchronous online program to improve T1D knowledge and perceived self‐efficacy among Italian school staff. The competencies acquired through the intervention contribute to creating a safer and more supportive school environment for children with T1D and strengthen staff readiness in responding appropriately to diabetes‐related needs.

## Funding

The authors have nothing to report.

## Ethics Statement

All procedures were authorized by the ethics committee of the authors (approval no. 4/01.03.2022). All procedures contributing to this work comply with the ethical standards of the relevant national and institutional committees on human experimentation and the Helsinki Declaration of 1975, as revised in 2008.

## Consent

Informed consent was obtained from all subjects involved in the study. All participants consented to the publication of de‐identified findings from this research.

## Conflicts of Interest

The authors declare no conflicts of interest.

## Supporting information


**Table S1:** Questions and their corresponding references.
**Table S2:** Socio‐demographic characteristics of the sample, comparison between individuals who completed and those who did not complete the pre‐ and the postassessment.

## Data Availability

The data that support the findings of this study are available from the corresponding author upon reasonable request.

## Endnotes

^1^
Glucagon is a drug approved for the management of acute hypoglycemia. It is authorized by the US Food and Drug Administration (FDA). In Italy, its use is approved by the Italian Medicines Agency (Agenzia Italiana del Farmaco, AIFA). Retrieved from: Morris CH, Baker J. Glucagon. [Updated 2025 Feb 6]. In: StatPearls [Internet]. Treasure Island (FL): StatPearls Publishing; 2025 Jan. Available from: https://www.ncbi.nlm.nih.gov/books/NBK559195.
