# Supplementary material for: Evaluation of the Impact of a Psychoeducational Program on Type 1 Diabetes in Italian Schools: A Pre–Post Study
Source: J Sch Health. 2026 Mar 30;96(5):e70141. doi: 10.1111/josh.70141 (PMC13034808; doi:10.1111/josh.70141)
Supplement: Supplementary file 1 — Table S1: Questions and their corresponding references. Table S2: Socio‐demographic characteristics of the sample, comparison between individuals who completed and those who did not complete the pre‐ and the postassessment. [file JOSH-96-0-s001.docx]

**Table S1.** Questions and their corresponding references

| n | English question | Italian question | Reference |
| --- | --- | --- | --- |
| **SECTION 1: Demographic and general information** | | | |
|  | Age: | La sua età |  |
|  | Gender:  a. Male  b. Female | Sesso:  a. Maschio  b. Femmina | N/A |
|  | Level of education  a. High school Graduation  b. Middle school Graduation  c. Degree  d. PhD or other post-laurea degree | Il suo livello di scolarità  a. Diploma scuola superiore  b. Diploma scuola media  c. Laurea specialistica/magistrale,  d. Titolo post-laurea (master, dottorato di ricerca) | N/A |
|  | Name of your school: | Il nome della scuola in cui lavora | N/A |
|  | Position in your school:  a. Principal  b. Teacher  c. Janitor | Ruolo rivestito a scuola:  a. Dirigente  b. Insegnante  c. Personale Ata | N/A |
|  | Type of school:  a. Public  b. Private | Tipo di scuola:  a. Pubblica  b. Privata | N/A |
|  | How many years have you been working in a school? | Da quanti anni lavora a scuola? | N/A |
|  | Do you have any diabetic student in your class?  a. Yes  b. No | Ha qualche alunno con diabete di tipo 1 nella sua classe?  a. Sì  b. No | Aycan et al., 2012 |
| **SECTION 2: Knowledge of Type 1 Diabetes** | | | |
| 1 | What is a diabetes disease?  a. A kidney disease  b. The body has not enough calcium  c. Lack of oxygen  d. The body has not enough insulin | Che cos’è il diabete di tipo 1?  a. Una patologia del rene  b. Una patologia a causa della quale il corpo non ha abbastanza calcio  c. Una mancanza di ossigeno  d. Una patologia a causa della quale il corpo non ha abbastanza insulina | Alzahrani, 2019 |
| 2 | Is diabetes a contagious disease?  a. Yes  b. No  c. I do not know. | Il diabete di tipo 1 è una patologia contagiosa?  a. Sì  b. No  c. Non so | Alzahrani, 2019 |
| 3 | What are the symptoms of diabetes?  a. Autism  b. Excessive thirst and urge to urinate  c. Bleeding and dizzy  d. High fever and sore throat | Quali tra i seguenti sono sintomi di diabete di tipo 1  a. Autismo  b. Sete estrema e bisogno continuo di fare pipì  c. Perdita di sangue e stordimento  d. Febbre alta e mal di gola | Alzahrani, 2019 |
| 4 | Type 1 diabetes is treated with:  a. A low-sugar diet  b. Insulin  c. Insulin and a low-sugar diet  d. I don’t know | Il diabete di tipo 1 si cura principalmente con  a. Una dieta povera di zuccheri  b. L’insulina  c. L’insulina associata a una dieta adeguata  d. Non so | Vespasiani et al., 2002 |
| 5 | What does hypoglycemia mean?  a. High blood sugar.  b. Low sugar in blood.  c. Sugar in urine.  d. I don’t know. | Ipoglicemia significa  a. Molto zucchero nel sangue  b. Poco zucchero nel sangue  c. Presenza di zucchero nelle urine  d. Non so | Vespasiani et al., 2002 |
| 6 | Which of the following are considered as low blood sugar sign?  a. Hot flashes, itching, diarrhea  b. Need to urinate, thirst, vomiting  c. Paleness, sweating, shakiness  d. I don't know | Quali tra i seguenti sono i segni più frequenti dell’ipoglicemia  a. Vampate di calore, prurito, diarrea  b. Bisogno di urinare, sete, vomito  c. Pallore, sudore freddo, tremore  d. Non so | Vespasiani et al., 2002 |
| 7 | Hypoglycemia could be handled at school  a. Yes, from teachers  b. Yes, only by 911  c. No  d. Yes, from the child with T1D and teachers | L’ipoglicemia può essere gestita a scuola  a. Sì, dagli insegnanti  b. Sì, solo dal 118  c. No  d. Sì, dal bambino stesso e dagli insegnanti | Bradbury and Smith, 1983; Gesteland and Lindsay, 1989 |
| 8 | What is the right procedure from the following when someone has a low blood sugar?  a. Give him/her glucose strip or sugar drink.  b. Give him/her a cup of water.  c. Give him/her chocolate.  d. Give him/her biscuits or sweets | Quale tra le seguenti è la procedura corretta da usare quando qualcuno ha livelli bassi di zucchero nel sangue  a. Dare bibita zuccherata o fialetta di glucosio  b. Dare una tazza di acqua  c. Dare un cioccolatino  d. Dare biscotti o dolci | Alzahrani, 2019 |
| 9 | Which of the following is considered as high blood sugar signs?  a. Frequent urination and thirstiness  b. Irritability  c. Hyperactivity  d. I don’t know. | Quale tra i seguenti si può considerare un segno di livelli elevati di zucchero nel sangue  a. Bisogno di urinare frequente e sete  b. Nervosismo  c. Iperattività  d. Non so | Alzahrani, 2019 |
| 10 | Is it necessary for a child/adolescent with type 1 diabetes to have a mid-morning snack?  a. Yes  b. No  c. I don't know | È necessario che un bambino/ragazzo con diabete di tipo 1 mangi uno spuntino a metà mattinata?  a. Sì  b. No  c. Non so | Bradbury and Smith, 1983 |
| 11 | A child/adolescent with type 1 diabetes at school should:  a. Avoid frequent blood glucose checks or eating snacks outside scheduled times  b. Participate fully in all school activities, with the freedom to self-monitor and consume necessary foods  c. Avoid participating in physical education classes  d. I don't know | Il bambino/ragazzo con diabete di tipo 1 a scuola deve  a. Evitare di fare molti controlli della glicemia o di fare lo spuntino fuori orario  b. Partecipare a tutta l’attività scolastica usufruendo della libertà di autocontrollarsi e di assumere gli alimenti necessari  c. Evitare di partecipare alle lezioni di educazione fisica  d. Non so | Bradbury and Smith, 1983; Gesteland 1989 |
| **SECTION 3: Attitude towards T1D, worries and beliefs on T1D** | | | |
| 1 | Diabetes has an impact on the student’s achievement  a. Yes  b. No  c. I don’t know | Il diabete di tipo 1 ha un impatto limitato sul rendimento degli studenti  a. Sì  b. No  c. Non so | Alzahrani, 2019 |
| 2 | Does a child/adolescent with type 1 diabetes miss more school than their classmates?  a. Yes  b. No  c. I don't know | Il bambino/ragazzo con diabete di tipo 1 fa più assenze dei suoi compagni  a. Sì  b. No  c. Non so | Gesteland and Lindsay, 1989 |
| 3 | I realize that diabetes can affect students' ability to pay attention in lessons.  a. Yes  b. No  c. I don't know | Mi rendo conto che il diabete di tipo 1 può influire sulla capacità degli studenti con tale patologia di prestare attenzione durante una lezione.  a. Sì  b. No  c. Non so | Alzahrani, 2019 |
| 4 | I consider diabetic students as special needs students  a. Yes  b. No  c. I don't know | Considero gli studenti con diabete di tipo 1 come studenti con bisogni speciali.  a. Sì  b. No  c. Non so | Alzahrani, 2019 |
| 5 | Children/adolescents with type 1 diabetes may have more behavioral problems  a. Yes  b. No  c. I don't know | I bambini/ragazzi con diabete di tipo 1 possono avere più problemi di comportamento  a. Sì  b. No  c. Non so | Gesteland and Lindsay, 1989 |
| 6 | Diabetic students make problems with their friends.  a. Yes  b. No  c. I don't know | Gli studenti con diabete di tipo 1 possono avere problemi con i loro amici  a. Sì  b. No  c. Non so | Alzahrani, 2019 |
| 7 | Do you think that more information about type 1 diabetes would improve children’s integration at school?  a. Yes  b. No  c. I don't know | Credo che avere maggiori informazioni sul diabete di tipo 1 migliorerebbero l'integrazione dei bambini a scuola  a. Sì  b. No  c. Non so | Gutzweiler et al., 2020 |
| **SECTION 4: Perceived competence about own knowledge of diabetes and in managing T1D** | | | |
| 1 | How would you personally feel about teaching a child with T1D?  Anxious, nervous  Relaxed  Confused  I don’t know | Come si sentirebbe personalmente nell'insegnare a un bambino con il diabete di tipo 1  a. Ansioso, preoccupato  b. Tranquillo  c. Confuso  d. Non so | Eiser et al., 2008 |
| 2 | In your opinion, is it helpful or reassuring for the parent to be able to monitor their child with type 1 diabetes from home and intervene at any time by calling and asking for corrections to be made?  a. Helpful, reassuring  b. Annoying, a source of anxiety  c. Intrusive  d. I don’t know | Secondo la sua opinione, il fatto che il genitore possa monitorare il bambino con diabete di tipo 1 da casa e in qualunque momento possa intervenire chiamando e chiedendo che vengano fatte correzioni è per lei  a. Utile, rassicurante  b. Fastidioso, fonte d’ansia  c. Invadente  d. Non so | n/d |
| 3 | How would you rate your knowledge about diabetes? (Rate from 1 to 10)  Very good (10)  Good  Fair  Poor (1) | Come giudica le sue conoscenze sul diabete di tipo 1  Molto buone (10)  Buone  Sufficienti  Scarse (1) | Gutzweiler et al., 2020 |
| 4 | How would you rate your ability in diabetes management?  Very good (10)  Good  Fair  Poor (1) | Come giudica le sue capacità di gestire il diabete di tipo 1  Molto buone (10)  Buone  Sufficienti  Scarse (1) | Gutzweiler et al., 2020 |
| 5 | How would you rate your ability to recognize symptoms of low blood glucose levels?  Very good (10)  Good  Fair  Poor (1) | Come giudica la sua capacità di riconoscere i sintomi di bassi livelli di zucchero nel sangue  Molto buona (10)  Buona  Sufficiente  Scarsa (1) | Gutzweiler et al., 2020 |
| 6 | How much do you think a parent of a child/adolescent with type 1 diabetes can interfere with the child’s school life?  A lot (10)  Quite a bit  A little  Not at all (1) | Quanto pensa possa interferire un genitore di un bambino/ragazzo con diabete di tipo 1 nella vita scolastica  Moltissimo (10)  Abbastanza  Poco  Per niente (1) | n/d |

**Table S2.** Sociodemographic characteristics of the sample, comparison between individuals who completed and those who did not complete the pre- and the post-assessment.

|  | Completed the educational program and the pre- and post-assesment  *N*=436  M(SD) | Did not complete the post-assessment  N=234  M(SD) | Completed the educational program and pre- and post-assessment vs. did not complete the post- assessment  test, p |
| --- | --- | --- | --- |
| **Gender** (female) (*n*) | 383 | 194 | 3.106, .078 |
| **Age** (years) *M(SD)* | 50.75 (± 10.03) | 51.08 (± 9.88) | -.415, .678 |
| **Year working in the school** M(*SD*) | 18.11 (± 11.5) | 18.17 (± 11.49) | -.068, .946 |
|  |  |  |  |
| **Level of education (n)** |  |  | 9.147, .027 |
| Middle School Graduation | 1 | 6 |  |
| High School Graduation | 99 | 59 |  |
| Degree | 300 | 148 |  |
| Post-lauream degree | 36 | 21 |  |
|  |  |  |  |
| **Position held in the school** (*n*) |  |  | 12.062, .007 |
| Principal | 4 | 1 |  |
| Teacher | 420 | 214 |  |
| Non-teaching staff | 11 | 19 |  |
| Not declared | 1 | 0 |  |
|  |  |  |  |
| **Type of school** (*n*) |  |  | .254, .614 |
| Public | 426 | 230 |  |
| Private | 10 | 4 |  |
|  |  |  |  |
| **Diabetic children in the class** (*n*) |  |  | .160, .689 |
| Yes | 275 | 132 |  |
| No | 161 | 72 |  |
|  |  |  |  |
| **Sum of answers part 2** *M (SD)* | 9.66 (± 1.35) | 9.52 (± 1.53) | 1.165, .245 |
|  |  |  |  |
